# A Theoretical Characterization of Curvature Controlled Adhesive Properties of Bio-Inspired Membranes

**DOI:** 10.3390/biomimetics1010003

**Published:** 2016-04-19

**Authors:** Luciano Afferante, Lars Heepe, Kirstin Casdorff, Stanislav N. Gorb, Giuseppe Carbone

**Affiliations:** 1 Department of Mechanics, Mathematics and Management, Politecnico of Bari, Bari 70126, Italy; giuseppe.carbone@poliba.it; 2 Department of Functional Morphology and Biomechanics, Zoological Institute, Kiel University, Kiel 24118, Germany; lheepe@zoologie.uni-kiel.de (L.H.); kcasdorff@ethz.ch (K.C.); sgorb@zoologie.uni-kiel.de (S.N.G.)

**Keywords:** adhesion, biomimetics, energy release rate, elastic membranes

## Abstract

Some biological systems, such as the tree frog, *Litoria caerulea*, and the bush-cricket, *Tettigonia viridissima*, have developed the ability to control adhesion by changing the curvature of their pads. Active control systems of adhesion inspired by these biological models can be very attractive for the development of devices with controllable adhesive properties. In this paper, we present a theory describing the adhesive behavior of an artificial system consisting of an inflatable membrane clamped to a metallic cylinder and filled with air. In such a system, by controlling the internal pressure acting on the membrane, it is possible to modulate the adhesive strength. In particular, an increase of the internal pressure and, hence, the curvature of the membrane, results in a decrease of the pull-off force. Results predicted by the theoretical model are in good agreement with experimental data. The model explains the apparent contradictory results observed for the thick membrane with zero curvature. In fact, in this case, large pull-off forces should be expected, but zero values are measured due to an initial small misalignment between indenter and membrane, which is not possible to control with precision during the experiments. The present model might help to achieve a better understanding of the adhesion behavior of biological systems and of the fingertips that, in a broad sense, may be regarded as shell-like structures.

## 1. Introduction

Adhesion is a fundamental phenomenon with great importance in all areas of life and technology, such as in biological systems (e.g., in cell [1] and bacterial [2] adhesion, plant [3] and animal [4] attachment), in technical systems (e.g., road-tire contact [5]), or simply using sticky tapes (e.g., Scotch^®^, Tesa^®^, Post-It^®^). The adhesive contact problem between a sphere and a plane has been solved theoretically by Bradley [6], Johnson, Kendall, and Roberts (JKR theory) [7], and Derjaguin, Müller, and Toporov (DMT theory) [8] for different limiting cases. Also, for other contact geometries, solutions have been proposed [9,10,11,12,13,14,15,16], e.g., peeling of a thin film from a rigid substrate [10,14]. Those theories have been successfully applied to many of the above mentioned adhesion problems both in science and technology. However, there are some biological systems which do not resemble the contact between a bulk sphere and a bulk plane. Instead, they consist of shell-like or vessel-like structures. Examples range from cells and bacteria to the smooth adhesive organs (toe pads) of the tree frog *Litoria caerulea* (Anura, Hylidae) [17] and that of the great green bush-cricket *Tettigonia viridissma* (Orthoptera, Tettigoniidae) [18]. For such systems, a different adhesive behavior is expected than those predicted by theories of solid bodies, e.g., JKR theory [19]. Such shell-like structures usually have a certain internal structure and are filled with fluids [17,18].

**Figure 1:**
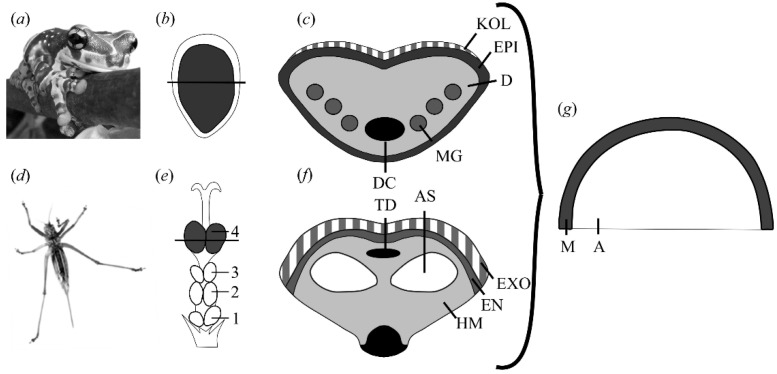
Comparison of the attachment pad of (**a**–**c**) a tree frog and (**d**–**f**) a bush-cricket. (**a**) Tree frog sticking to a branch. (**b**) Ventral view of the toe pad of the tree frog *Litoria caerulea*. (**c**) Cross section of the adhesive pad of *L. caerulea*. D, dermis; DC, digital cartilage; EPI, epidermis; KOL, keratinised outer layer; MG, mucous gland; (strongly schematized after [12]). (**d**) Bush-cricket sticking to the glass. (**e**) Ventral view of the tarsus of *Tettigonia viridissima* with four tarsomeres (1–4); (**f**) Cross section of the adhesive pad of *T. viridissima*. AS, air sack; EN, endocuticle; EXO, exocuticle; HM, haemolymph; TD, tendon of the claw flexor muscle (adapted from [13] with modifications). (**g**) Abstracted model of (**c**) and (**f**). A, air; M, membrane. Adapted from [20].

By varying the amount of fluid or varying the internal pressure of such shell-like structures for the tree frog and the bush-cricket, it has been speculated that those animals can actively control the curvature of their adhesive organ and thus the adhesion to the substrate (see Figure 1) [20]. Using an artificial model system (Figure 1g) inspired by those animals, the hypothesis of adhesion control by inflation was tested [20]. Indeed, a curvature controlled adhesion was observed, but a different adhesion behavior compared to the JKR theory was observed. In addition, related works reported in the literature [21,22,23,24,25,26,27,28,29,30,31], dealing with similar configurations, do not capture our problem on the whole. For example, the adhesion of a flat punch on a thin pre-stressed membrane, but in the absence of pressure loads, is analyzed in [26]. In [28], instead, the problem of a pressurized membrane immersed in water is addressed, but prestrain effects are neglected. Moreover, the approach given in [22,27] assumes: (i) circumferential and meridian strains equal and constant in the membrane; and (ii) small displacements. These assumptions do not entirely fit to our situation, for example, at low pressure loads the circumferential strains are negligible with respect to the meridian ones.

Thus, the aim of the present study is to develop a theoretical model that captures all aspects of the experimental work done in [20] by reducing the number of necessary assumptions.

This paper is organized as follows: In Section 2 the experimental setup and the tests carried out on the artificial model are briefly reviewed. In Section 3 the theory is introduced. The results of the theory are presented in Section 4 and discussed in the context of the experimental data obtained in [20]. Finally, Section 5 concludes the study discussing the biological and technological relevance of curvature controlled adhesion.

## 2. Materials and Methods

**Figure 2:**
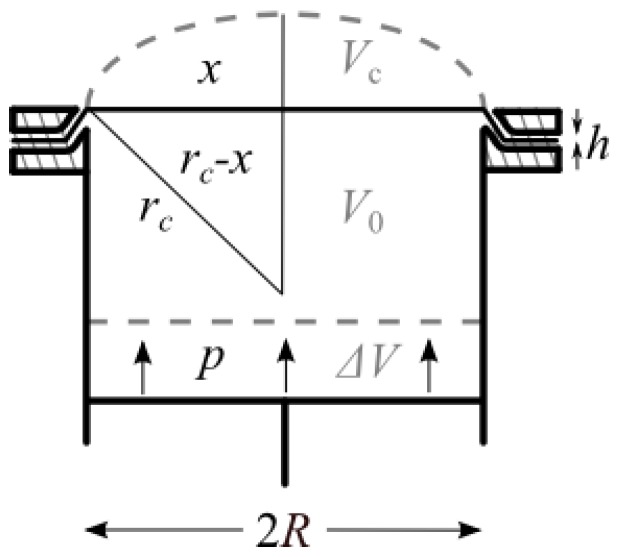
A hollow cylinder with a membrane fixed at one side and having a piston at the other side. Dashed lines show the state after pressurization. *h*, membrane thickness; *p*, uniform pressure; *r*_c_, radius of curvature; *R*, base radius; Δ*V*, volume difference; *V*_0_, initial volume; *V*_c_, cap volume; *x*, maximum height. Not to scale. Reprinted from [20] with permission. Copyright Springer 2014.

Figure 2 shows schematically the artificial system mimicking the smooth adhesive organs of the tree frog and the bush-cricket (see Figure 1). Membranes made of polyvinylsiloxane (PVS) were clamped at one side of a hollow cylinder.

The diameter 2*R* of the cylinder was 20 mm. By changing the volume of the cylinder with a piston pneumatically different curvatures of the membranes could be adjusted. Membranes with average thickness of ~145 μm, and ~270 μm were used in the experiments. Also, an average RMS-roughness of 0.116 μm (± 0.017 μm, *n* = 10) was measured using a white light interferometer (WLI) NewView 6k (ZygoLOT GmbH, Darmstadt, Germany). Although biological attachment systems mainly operate due to hydraulic mechanisms, we here used a pneumatic approach as those hydraulic systems are not closed systems, but open ones and thus those systems very likely operate as compressible systems.

**Figure 3:**
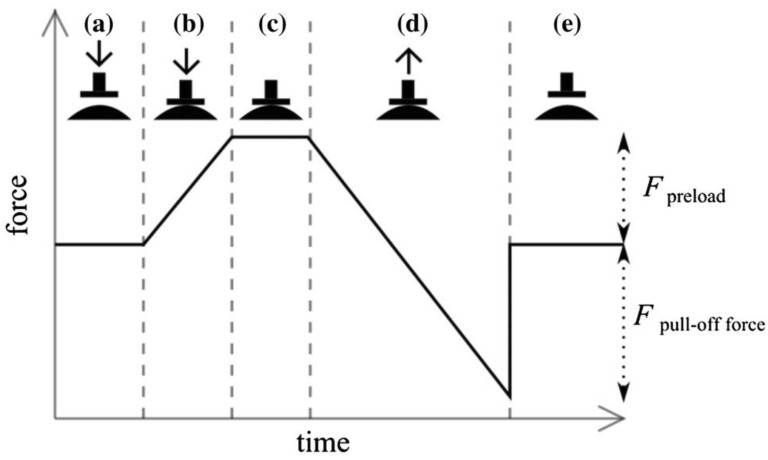
A typical force-time sequence of adhesion measurement: (**a**,**b**) preloading the membrane with an indenter at constant velocity until a certain load is reached; (**c**) after a resting phase the indenter is withdrawn from the membrane until complete detachment (**d**,**e**). Reprinted from [20] with permission. Copyright Springer 2014.

Pull-off forces were measured by preloading membranes at different radii of curvatures with a flat circular glass indenter (diameter 10 mm, RMS-roughness 0.003 μm (± 0.001 μm, *n* = 5)). Figure 3 shows the typical force-time sequence of pull-off measurements. The indenter was lowered towards the membrane until a fixed preload of 3 mN was reached (Figure 3a,b). After a resting phase of ~2 s (Figure 3c), the indenter was withdrawn from the membrane (Figure 3d) until it was finally detached (Figure 3e).

## 3. Theoretical Model

Consider an elastic membrane clamped at the external radius *R* as schematically shown in Figure 2. By reducing the initial volume of the system *V_0_* by Δ*V*, an increase of the initial atmospheric pressure *p*_0_ occurs. In addition, the volume reduction results in a deformation of the membrane and produces an extra cap volume *V*_c_ < Δ*V*. Assuming isothermal conditions, the resulting pressure increase Δ*p* can be calculated from the Boyle–Mariotte law as

(3.1) $$p_{0}+\Delta p=p_{0}\frac{V_{0}}{V_{0}-\left[\Delta V-V_{c}\left(\Delta p\right)\right]}$$

where *V*_c_ is obtained assuming a spherical cap with the curvature radius *r_c_* of the inflated membrane. The radius *r_c_*, in turn, can be experimentally determined for each membrane and volume reduction Δ*V* as described in [20].

When the plate indenter adhering to the membrane is pulled up, an additional variation in volume occurs. However, such variation is several orders of magnitude smaller than the control volume of the apparatus. As a result, the resulting variation in pressure occurring during the detachment of the plate will be negligible.

For a reversible and isothermal transformation, the total energy of the system (*U_tot_*) is the sum of the strain energy (*U_el_*), the potential energies associated with the applied force *F* (*U_P_*) and pressure Δ*p* (*U*_Δ*p*_), and the surface energy (*U_ad_*). When the variation of the total energy is negative, the system spontaneously moves out of equilibrium. Therefore, detachment spontaneously occurs when

(3.2) $$dU_{tot}=dU_{el}+dU_{P}+dU_{\Delta p}+dU_{ad}\leq 0$$

where *dU_el_* is the change in the elastic energy stored in the membrane, *dU_P_* and *dU*_Δ*P*_ are the variation of the potential energy associated with the applied detachment force *F* and pressure Δ*p*, respectively, and *dU_ad_* = −Δγ*dA* is the change in the surface energy, being *A* the contact area between the solid indenter and the membrane and Δγ the so-called work of adhesion per unit area.

Recalling that the energy release rate *G* at the tip of the contact area is defined as in [32]

(3.3) $$G=\frac{\partial \left(U_{tot}-U_{ad}\right)}{\partial A}=\frac{\partial U_{el}}{\partial A}+\frac{\partial U_{P}}{\partial A}+\frac{\partial U_{\Delta p}}{\partial A}$$

the system spontaneously moves out of equilibrium when

(3.4) $$dU_{tot}=\left(G-\Delta \gamma \right)dA\leq 0$$

If *G* > Δγ the above inequality requires *dA* < 0 and hence the contact area reduces causing the detachment of the membrane. *Vice versa*, if *G* < Δγ the detachment is prevented. Therefore, the contact area at the equilibrium can be evaluated by enforcing the condition *G* = Δγ.

The change in the potential energies *U_P_* and *U*_Δ*p*_ occurring for a variation of the contact area *d*A = 2π*ada* is, respectively,

(3.5) $$dU_{P}=-F\cdot du\left(a\right)=-F\left[u\left(a+da\right)-u\left(a\right)\right]$$

(3.6) $$\begin{array}{ll} dU_{\Delta p} & =-\Delta p\cdot dV\left(a\right)=-\Delta p\left[V\left(a+da\right)-V\left(a\right)\right]\\ & =-\Delta p\left[\pi a\left(a+2da\right)u\left(a+da\right)-\pi a^{2}u\left(a\right)+2\pi \left(\int_{a+da}^{R}u_{a+da}\left(r\right)rdr-\int_{a}^{R}u_{a}\left(r\right)rdr\right)\right] \end{array}$$

with *a* being the contact radius and *u* the vertical displacement of the membrane. Notice *u* is a function of the radial position *r* and also depends on the value of the contact radius. In particular, *u_a_*(*a*) and *u*_a+da_(*a* + *da*) denote the values that the vertical displacement takes at *r* = *a* and *r* = *a* + *da*, respectively, when the contact radius is currently *a* or *a* + *da*.

In order to calculate *u* and the change in the elastic energy with the contact area, *dU_el_*, Finite Element (FE) calculations have been carried out with the aid of the software ANSYS. In particular, axisymmetric linear elastic shell elements have been adopted. The contact zone between the flat rigid indenter and the adhesive membrane is considered in terms of sticking friction, as suggested by some experiments [33]. This condition is taken into account assuming zero radial displacement in such a zone. Finite strain effects are also included by performing a large deflection analysis.

Since we assume linear elastic behavior, the energy stored in the membrane is not affected by the load history. Therefore, to calculate the change in total energy occurring during the process of detachment, at given applied force *F*, the following procedure has been implemented: (i) the radius of contact *a*_i_ between membrane and indenter is fixed; (ii) the load pressure Δ*p* and the vertical force *F*, on the node lying on the radius *a*_i_, are applied; (iii) the vertical *u*(*r*) and radial *v*(*r*) displacements are calculated; (iv) the corresponding stress and strain fields are evaluated; (v) the elastic energy *U*_el_ stored in the system is calculated; then (vi) the procedure is repeated for a new value of the contact radius *a*_i+1_ = *a*_i_ + Δ*a*.

The energy release rate *G*_i_ corresponding to the contact radius *a*_i_ is hence estimated as

(3.7) $$G_{i}=\frac{\Delta U_{tot,i}}{\Delta A_{i}}-\Delta \gamma =\frac{\left(U_{el_{i+1}}-U_{el_{i}}\right)+\left(U_{P_{i+1}}-U_{P_{i}}\right)+\left(U_{\Delta p_{i+1}}-U_{\Delta p_{i}}\right)}{2\pi a_{i}\Delta a}$$

The value *a* of the contact radius at the equilibrium is then obtained from the condition *G* = Δγ. The unstable detachment of the rigid plate from the membrane occurs at the saddle point of the total energy of the system, *i.e.,* when the minimum of the energy release rate *G*_min_ equals the work of adhesion Δγ. Therefore, to calculate the critical detachment force (the pull-off force *F*_pull-off_), we need to enforce the condition *G*_min_ = *Δ*γ.

## 4. Results and Discussion

Calculations are performed for the volume reductions considered in the experiments in [20]. The corresponding increases in pressure Δ*p* are averaged over all measured values. Since an unknown prestrain is introduced in the membrane when it is fixed on the tube, the stored strain energy *U*_el_ will also depend on the initial state of prestrain ε°_ij_ (and prestress σ°_ij_) of the membrane. An estimation of ε°_ij_ can be done assuming the prestrain uniform in the radial (ε°_r_) and circumferential (ε°_θ_) direction and calculating its values by tuning the pull-off force obtained for the highest Δ*p* on the experimental data. Calculations at the other levels of pressure are hence performed assuming negligible changes in prestrain with the volume reduction.

**Figure 4:**
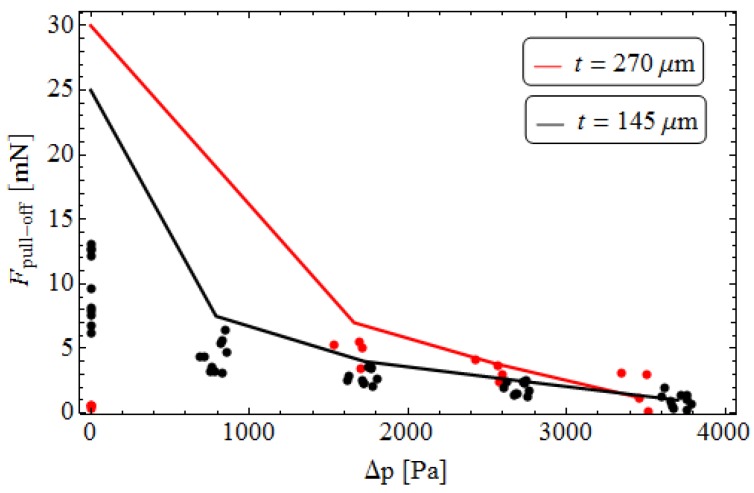
The theoretically calculated pull-off force *F*_pull-off_ in terms of the pressure increase Δ*p* for different membrane thicknesses (*t* = 145 μm, 270 μm). Experimental data given in [20] are also plotted for comparison.

Figure 4 shows the theoretically calculated pull-off force *F*_pull-off_ as a function of the pressure increase Δ*p*, corresponding to the volume reductions of 1, 2, 3 and 4 mL, for two different membrane thicknesses (*t* = 145 μm, 270 μm). Calculations are carried out assuming a work of adhesion Δγ = 50 mJ/m^2^, which is really an equivalent average value, experimentally measured on nominally “flat” surfaces. The experimental data, given in [20], are also plotted for comparison.

Results show that the pull-off force required to detach the indenter increases with decreasing the pressure Δ*p* and, hence, with increasing the radius of curvature. Furthermore, *F*_pull-off_ is larger for the “thick” membrane because the latter deforms less and hence stores less elastic energy, according to the experimental results given in [34]. A sufficiently good agreement between theoretical results and experimental data of Dening *et al.* [20] can be noticed, with some differences for the lower levels of pressure (corresponding to the higher radii of curvature of the membrane). These differences probably occur because, in the experimental tests, the distribution of prestrain in the membrane is not properly uniform.

Moreover, the significant difference in results for flat membrane (Δ*p* → 0 and *r*_c_ → ∞) between numerical model and experimental tests is probably due to a small misalignment between indenter and membrane. Such an assumption also gives a reasonable explanation to the completely different behavior at infinite *r*_c_ of the “thin” and “thick” membrane used in the experiments. Due to the much higher bending stiffness (scales with *h*^3^) of the thick membrane, in [20] it was speculated that a certain degree of misalignment between indenter surface and thick membrane prevent the formation of complete contact. In fact, the “thin” membrane can anyway completely or nearly attach to the non-parallel indenter surface, resulting in high pull-off forces. In contrast, the “thick” one is not able to attach to the indenter, since the gain in adhesion energy would be smaller than the work to be done to bring the membrane into complete contact, resulting in vanishing pull-off forces.

**Figure 5:**
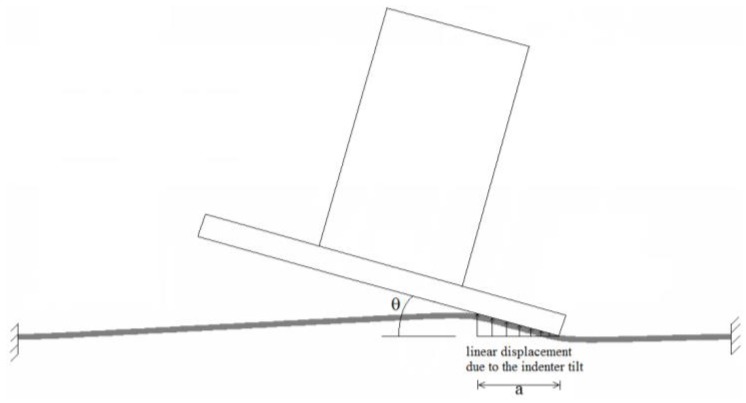
The scheme of the model adopted to investigate qualitatively the effects of the indenter tilting on the adhesion behavior of the membrane.

For flat membranes (*i.e.*, when Δ*p* = 0), it has been shown in [35,36] that the circumferential strains and stresses negligibly affect the strain energy stored in the material. For this reason, with the aim to qualitatively understand how the tilt angle of the plate indenter affects the adhesion behavior, we have performed calculations on the simplified 2D plain-strain system schematically shown in Figure 5. The scope is to verify if a small tilt angle of the plate indenter can determine a significant reduction in the pull-off force.

Let us assume membrane deformation is due only to the indenter tilting, for properly evaluating its effect on the initial contact size. In all cases, the same initial value of prestrain and a work of adhesion Δγ = 50 mJ/m^2^ are been considered. In order to determine, for a given tilt angle, the contact size *a* at equilibrium, we need to calculate the value of *a* at which the total energy *U_tot_* of the system takes a minimum. For this purpose, we initially assume the value of *a*, and then deform the membrane by applying on the contact region the corresponding displacements field (see Figure 5). Such operation is repeated for different values of *a*, so the total energy is determined as a function of the contact size. To calculate *U_tot_* the same approach as that described in Section 3 is used. However, since, in such case, the system is displacement controlled, the only energetic terms that we need to consider are the elastic energy stored in the membrane and the adhesion one.

**Figure 6:**
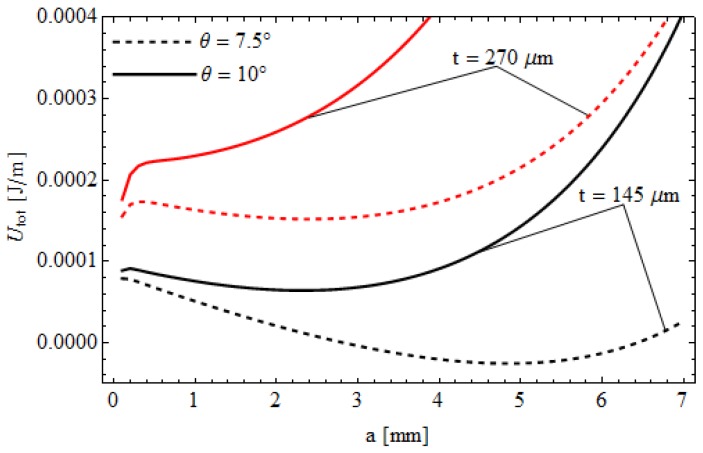
The total energy *U_tot_* of the system, shown in Figure 3, as a function of the contact size *a*, for different tilting angles (θ = 7.5°, 10°) and membrane thicknesses (*t* = 145 μm, 270 μm).

Example results are summarized in Figure 6, where the total energy *U_tot_* of the system is shown as a function of the contact size *a*, for different tilting angles and membrane thicknesses. When θ = 10° and *t* = 270 μm (red solid line), *U_tot_* has a minimum at *a* = 0. As a result, adhesion between indenter and membrane may not occur. Similarly, for *t* = 145 μm (black solid line), we have a minimum at *a* > 0 and, hence, a positive pull-off force will be necessary to detach the indenter. At smaller indenter tilt (θ = 7.5°) adhesion is possible also when *t* = 270 μm (red dashed line), because the minimum of *U_tot_* occurs at *a* > 0. However, the contact size is smaller than the one occurring at *t* = 145 μm (black dashed line). Such results confirm our hypothesis that the vanishing pull-off force measured in the experimental tests on the “thick” membrane at *r*_c_ → ∞ (*i.e.*, Δ*p* = 0) are probably due to an initial small misalignment between indenter and membrane. Moreover, the above described behavior, could also explain the differences observed at low Δ*p* between experimental data and theoretical predictions.

## 5. Conclusions and Outlook

We have presented a theoretical model for the adhesion of an elastic membrane with different curvatures in contact with a flat rigid indenter. Good quantitative agreement with the experimental results is obtained. The effect of the membrane thickness and the role of small misalignment between indenter surface and membrane have been investigated. In combination with the results obtained in [20] the present work now provides a more coherent picture of the contact mechanics of curvature controlled adhesion of shell-like structures and may, thus, allow a better understanding of the adhesion behavior of shell-like structures in biological systems such as the smooth attachment organs of tree frogs and bush-crickets. Moreover, it may also be applicable to the field of perception of surfaces, haptics, and tactile sense by human fingertips [37,38,39]. Fingertips may also be regarded as shell-like structures. Haptics of product surfaces increasingly become an important product feature, and a thorough understanding of human perception of surfaces depending on certain surface and material properties is crucial [40]. One important property is the adhesion and friction of fingertips on surfaces and this model might help us to obtain a better understanding of this [39,41]. Moreover, this model might be the basis for an optimal design of new curvature controlled, energy efficient gripping solutions as proposed in [20].

## References

[1] Gumbiner B.M. (1996). Cell adhesion: The molecular basis of tissue architecture and morphogenesis. Cell.

[2] Tsang P.H., Li G., Brun Y.V., Freund L.B., Tang J.X. (2006). Adhesion of single bacterial cells in the micronewton range. Proc. Natl. Acad. Sci. USA.

[3] Barthlott W., Neinhuis C. (1997). Purity of the sacred lotus, or escape from contamination in biological surfaces. Planta.

[4] Gorb S.N. (2001). Attachment Devices of Insect Cuticle.

[5] Persson B.N.I. (2000). Sliding Friction: Physical Principles and Applications.

[6] Bradley R.S. (1932). LXXIX. The cohesive force between solid surfaces and the surface energy of solids. Philos. Mag..

[7] Johnson K.L., Kendall K., Roberts A.D. (1971). Surface energy and the contact of elastic solids. Proc. R. Soc. A.

[8] Derjaguin B.V., Muller V.M., Toporov Y.P. (1975). Effect of contact deformations on the adhesion of particles. J. Colloid Interface Sci..

[9] Kendall K. (1971). The adhesion and surface energy of elastic solids. J. Phys. D Appl. Phys..

[10] Kendall K. (1975). Thin-film peeling-the elastic term. J. Phys. D Appl. Phys..

[11] Spolenak R., Gorb S., Gao H., Arzt E. (2005). Effects of contact shape on the scaling of biological attachments. Proc. R. Soc. A.

[12] Afferrante L., Ciavarella M., Demelio G. (2015). Adhesive contact of the Weierstrass profile. Proc. R. Soc. A.

[13] Afferrante L., Carbone G. (2012). Biomimetic surfaces with controlled direction-dependent adhesion. J. R. Soc. Interface.

[14] Putignano C., Afferrante L., Mangialardi L., Carbone G. (2014). Equilibrium states and stability of pre-tensioned adhesive tapes. Beilstein J. Nanotechnol..

[15] Carbone G., Pierro E., Gorb S. (2011). Origin of the superior adhesive performance of mushroom-shaped microstructured surfaces. Soft Matter.

[16] Afferrante L., Grimaldi G., Demelio G., Carbone G. (2015). Direction-dependent adhesion of micro-walls based biomimetic adhesives. Int. J. Adhes. Adhes..

[17] Barnes W.J.P., Goodwyn P.J.P., Nokhbatolfoghahai M., Gorb S.N. (2011). Elastic modulus of tree frog adhesive toe pads. J. Comp. Physiol. A.

[18] Gorb S., Jiao Y., Scherge M. (2000). Ultrastructural architecture and mechanical properties of attachment pads in *Tettigonia Viridissima* (Orthoptera Tettigoniidae). J. Comp. Physiol. A.

[19] Shi J., Müftü S., Wan K.T. (2011). Adhesion of an Elastic Convex Shell onto a Rigid Plat. J. Adhes..

[20] Dening K., Heepe L., Afferante L., Carbone G., Gorb S.N. (2014). Adhesion control by inflation: Implications from biology to artificial attachment device. Appl. Phys. A.

[21] Gent A.N., Lewandowski L.H. (1987). Blow-off pressures for adhering layers. J. Appl. Polym. Sci..

[22] Wan K.T., Mai Y.W. (1995). Fracture-mechanics of a new blister test with stable crack-growth. Acta Metal. Mater..

[23] Williams J.G. (1997). Energy release rates for the peeling of flexible membranes and the analysis of blistrer tests. Int. J. Fract..

[24] Wan K.T. (2001). Adherence of an axisymmetric flat punch on a flexible membrane. J. Adhes..

[25] Wan K.T. (2002). Adherence of an axisymmetric flat punch onto a clamped circular plate: Transition from a rigid plate to a flexible membrane. J. Appl. Mech..

[26] Wan K.T., Dillard D.A. (2003). Adhesion of a flat punch adhered to a thin pre-stressed membrane. J. Adhes..

[27] Raegen A.N., Dalnoki-Veress K., Wan K.T., Jones R.A.L. (2006). Measurement of adhesion energies and Young's modulus in thin polymer films using a novel axi-symmetric peel test geometry. Eur. Phys. J. E.

[28] Flory A.L., Brass D.A., Shull K.R. (2007). Deformation and adhesive contact of elastomeric membranes. J. Polym. Sci. Part B.

[29] Point N., Sacco E. (1996). A delamination model for laminated composites. Int. J. Solids Struct..

[30] Point N., Sacco E. (1996). Delamination of beams: An application to DCB specimen. Int. J. Fract..

[31] Bretelle A.-S., Cocou M., Monerie Y. (2001). Unilateral contact with adhesion and friction between two hyperelastic bodies. Int. J. Eng. Sci..

[32] Maugis D. (1999). Contact, Adhesion and Rupture of Elastic Solids, Springer Series in Solid State Sciences.

[33] Varenberg M., Gorb S. (2007). Shearing of fibrillar adhesive microstructure: friction and shear-related changes in pull-off force. J. R. Soc. Interface.

[34] Song S., Sitti M. (2014). Soft Grippers Using Micro-fibrillar Adhesives for Transfer Printing. Adv. Mater..

[35] Afferrante L., Carbone G., Demelio G., Pugno N. (2013). Adhesion of Elastic Thin Films: Double Peeling of Tapes Versus Axisymmetric Peeling of Membranes. Tribol. Lett..

[36] Afferrante L., Carbone G. (2013). The mechanisms of detachment of mushroom-shaped micro-pillars: From defect propagation to membrane peeling. Macromol. React. Eng..

[37] Tomlinson S.E., Lewis R., Carré M.J. (2007). Review of the frictional properties of finger-object contact when gripping. Proc. Inst. Mech. Eng. J.

[38] Adams M.J., Johnson S.A., Lefèvre P., Lévesque V., Hayward V., André V., Thonnard J.-L. (2013). Finger pad friction and its role in grip and touch. J. R. Soc. Interface.

[39] van Kuilenburg J., Masen M.A., van der Heide E. (2013). A review of fingerpad contact mechanics and friction and how this affects tactile perception. Proc. Inst. Mech. Eng. J.

[40] Wongsriruksa S., Howes P., Conreen M., Miodownik M. (2012). The use of physical property data to predict the touch perception of materials. Mater. Des..

[41] Spinner M., Wiechert A.B., Gorb S.N. (2016). Sticky fingers: Adhesive properties of human fingertips. J. Biomech..

